# Speed and Accuracy of Visual Motion Discrimination by Rats

**DOI:** 10.1371/journal.pone.0068505

**Published:** 2013-06-28

**Authors:** Pamela Reinagel

**Affiliations:** Section of Neurobiology, Division of Biological Sciences, University of California San Diego, La Jolla, California, United States of America; Australian National University, Australia

## Abstract

Animals must continuously evaluate sensory information to select the preferable among possible actions in a given context, including the option to wait for more information before committing to another course of action. In experimental sensory decision tasks that replicate these features, reaction time distributions can be informative about the implicit rules by which animals determine when to commit and what to do. We measured reaction times of Long-Evans rats discriminating the direction of motion in a coherent random dot motion stimulus, using a self-paced two-alternative forced-choice (2-AFC) reaction time task. Our main findings are: (1) When motion strength was constant across trials, the error trials had shorter reaction times than correct trials; in other words, accuracy increased with response latency. (2) When motion strength was varied in randomly interleaved trials, accuracy increased with motion strength, whereas reaction time decreased. (3) Accuracy increased with reaction time for each motion strength considered separately, and in the interleaved motion strength experiment overall. (4) When stimulus duration was limited, accuracy improved with stimulus duration, whereas reaction time decreased. (5) Accuracy decreased with response latency after stimulus offset. This was the case for each stimulus duration considered separately, and in the interleaved duration experiment overall. We conclude that rats integrate visual evidence over time, but in this task the time of their response is governed more by elapsed time than by a criterion for sufficient evidence.

## Introduction

Often animals must commit to actions despite unresolved uncertainty. Taking more time to gather evidence can improve certainty about outcome. But taking too much time to gather information can be costly: time is a limited resource, and action can be time-critical. All animals therefore need mechanisms to balance the cost of waiting for more information against its potential value in a given context. When sensory information is limiting, how do animals determine the time of action? This question has been studied extensively for visually guided decisions in humans and non-human primates [1]–[19]. More recently, the temporal integration of olfactory or auditory information to guide behavioral choice has been studied in rodents [20]–[24].

Little is known, however, about the capacity of rats to integrate ongoing visual information to efficiently guide survival-relevant choices. Therefore we adapted a classic visual stimulus from the primate literature – the coherent random dot motion stimulus [1], [25]–[27] – to study the visual guidance of action in rats. We constructed an environment in which rats earned all their water by discriminating the direction of visual motion on a computer display. The motion direction revealed which of two water ports would dispense water – and which would cause a time-out during which no water could be earned. Other than these time-out periods, rats could trigger a motion stimulus at any time, and were then free to watch the motion stimulus for any length of time before committing to one of the two water ports. The strength of the motion signal was varied from nonexistent (0% coherence) to highly salient (100% coherence), allowing us to probe whether and how rats adapt to the reliability of the available visual information.

## Results

We trained six rats to discriminate the direction of visual motion in a coherent random dot motion display. In each trial, a field of 100 dots appeared at random locations on the display and immediately began to move. A subset of the dots drifted coherently either to the left or right, indicating the location of the rewarded response port. The remaining dots moved in random directions. The time of stimulus onset was controlled by the rat, which requested a trial by licking a port at the bottom center of the display. The time of the response was also controlled by the rat, which could terminate a trial at any time by licking either the left or right response port. This response triggered the stimulus offset. If the response was correct, a small liquid reward was delivered immediately, and the rat could initiate a new trial at any time. If the response was incorrect, no reward was delivered and a time-out was imposed before a new trial could be initiated. The duration of the timeout was fixed within each experiment, and ranged from 2–8 s for different experiments and subjects. After training to steady state performance, we analyzed the reaction times of error trials and correct trials.

**Figure 1:**
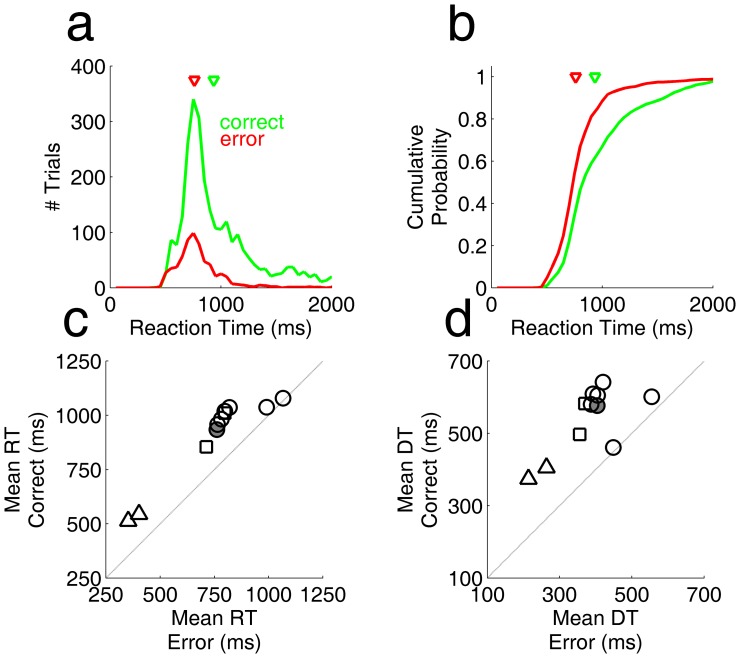
Correct trials have longer reaction times than error trials. **a.** Distribution of reaction times for one rat in a block with fixed (85%) coherence, for correct responses (green) and error trials (red). Arrows indicate the mean reaction times. **b.** Cumulative probability distributions of same data shown in panel a. Arrows indicate mean reaction times. **c.** Mean reaction time of error trials vs. correct trials across 11 experiments from 6 rats. Stimulus parameters were fixed across trials within each experiment, but differed between experiments. Motion coherence was 85% (circles, triangles) or 95% (squares). Response ports were either 90 mm (circles, squares) or 10 mm (triangles) from the central trial-initiation port. Shaded symbol indicates the example experiment of panels a-b. **d.** Mean estimated decision time of error trials vs. correct trials, for the same data analyzed in c. Decision time is defined as the reaction time in each trial minus each rat’s minimum reaction time over all trials.

First we asked whether the reaction time distribution differed between error trials and correct trials. Although no time limit was imposed, nearly all trials were completed within 2 seconds. We found that correct trials were more likely to have long reaction times, as shown for a typical example experiment in Figure 1a. This shift in reaction time distribution is easily visualized as a rightward shift in the cumulative probability of reaction times (Figure 1b). The result from one such experiment can be summarized by the mean reaction time of error trials and correct trials (arrows in Figure 1a and b). Similar results were found for all subjects and stimulus parameters tested (Figure 1c; P<10^−3^, Wilcoxon sign rank test). On average, correct responses took 24±12% more time than the error trials in the same experiment (mean±SD; range 1%–45% across subjects and experiments).

Each rat had a characteristic minimum response latency, which was invariant over the course of learning and insensitive to task parameters. If we take this as an estimate of the subject’s visual latency and motor delay, then the “decision time” can be defined as reaction time minus this minimum latency (Figure 1d). The decision time for correct trials was 44±21% longer than error trials (range 3–75% across subjects and experiments).

**Figure 2:**
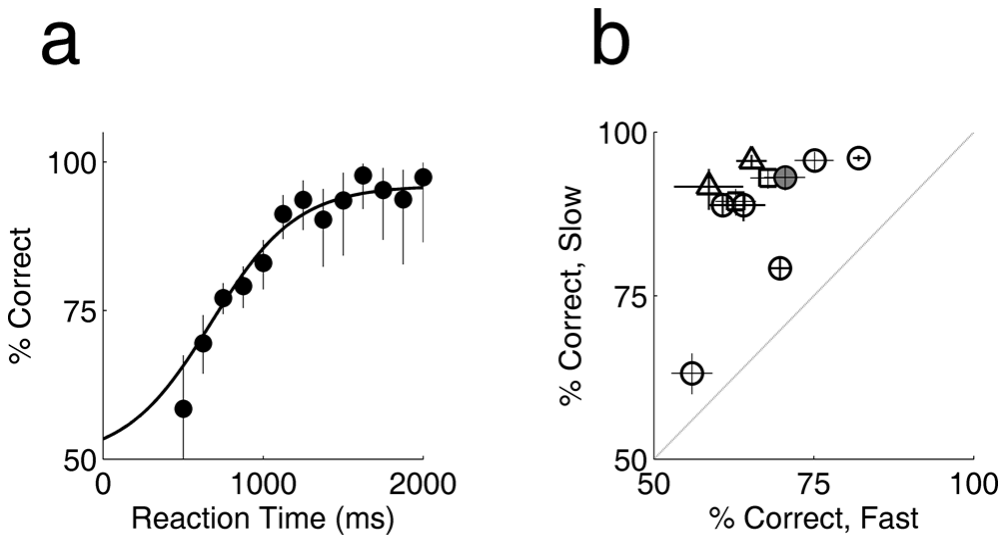
Accuracy increases with reaction time. **a.** Accuracy (% correct) as a function of reaction time, from same data as Figure 1 a–b. Error bars indicate binomial confidence intervals. **b.** Accuracy (% correct) is higher in slow trials than fast trials across the population (11 experiments from 6 rats; symbols defined in Figure 1c). Fast and slow trials are defined as the highest and lowest quartile of each rat’s overall RT distribution within the block respectively. Crosses indicate binomial confidence intervals.

Given that correct trials have longer reaction times than errors, the probability of being correct must increase with reaction time. On further analysis, we found that accuracy increased with reaction time from about 500–1200 ms, as shown for one experiment in Figure 2a. Accuracy approached 100% for reaction times above 1 second. Although overall accuracy and average speed varied widely across experiments and rats, in every case the slowest responses were more accurate than the fastest (Figure 2b; P<10^−3^, Wilcoxon sign rank test).

Accuracy improved with response latency when coherence was fixed across trials (Figures 1 and 2). We wondered whether the strength of the motion signal would modulate rats’ reaction times on a trial-by-trial basis. In a new set of experiments, coherence was selected independently for each trial from a broad, uniform probability distribution.

**Figure 3:**
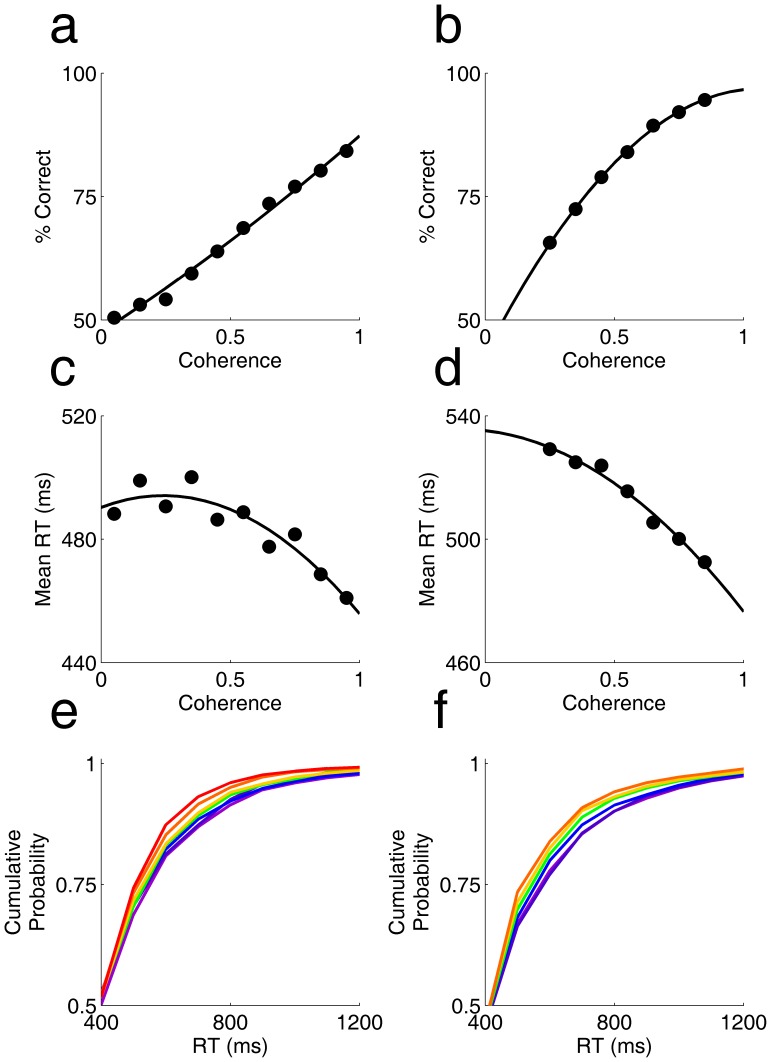
Dependence of accuracy and reaction time on motion strength. **a.** Psychometric function – accuracy (% correct) as a function of motion strength (coherence) when coherence was varied from 0–1 in randomly interleaved trials. Symbols indicate data; curve is a 2nd order polynomial fit. **b.** Psychometric function from another experiment in which coherence was drawn uniformly from the interval 0.2–0.9. **c.** Chronometric function – mean reaction time as a function of coherence, for the experiment of panel a. **d.** Chronometric function for the experiment of panel b. **e.** Cumulative probability of reaction times for different coherence ranges, for experiment of panel a. Color indicates motion coherence from low (cool) to high (hot). Cumulative probability was computed over the range of 0–3 s; axis is expanded to focus on the time range of interest. **f.** Cumulative probability of reaction times for experiment of panel b. Color scale same as in panel e.

We verified that rats’ accuracy in judging direction of motion improved with motion coherence over the range of values tested (Figure 3 a,b). We found a small but systematic decrease in mean reaction time as coherence increased (Figure 3 c,d). The difference arose from an increase in the tail of longer reaction times; the median reaction time was unchanged (Figure 3 e,f).

**Figure 4:**
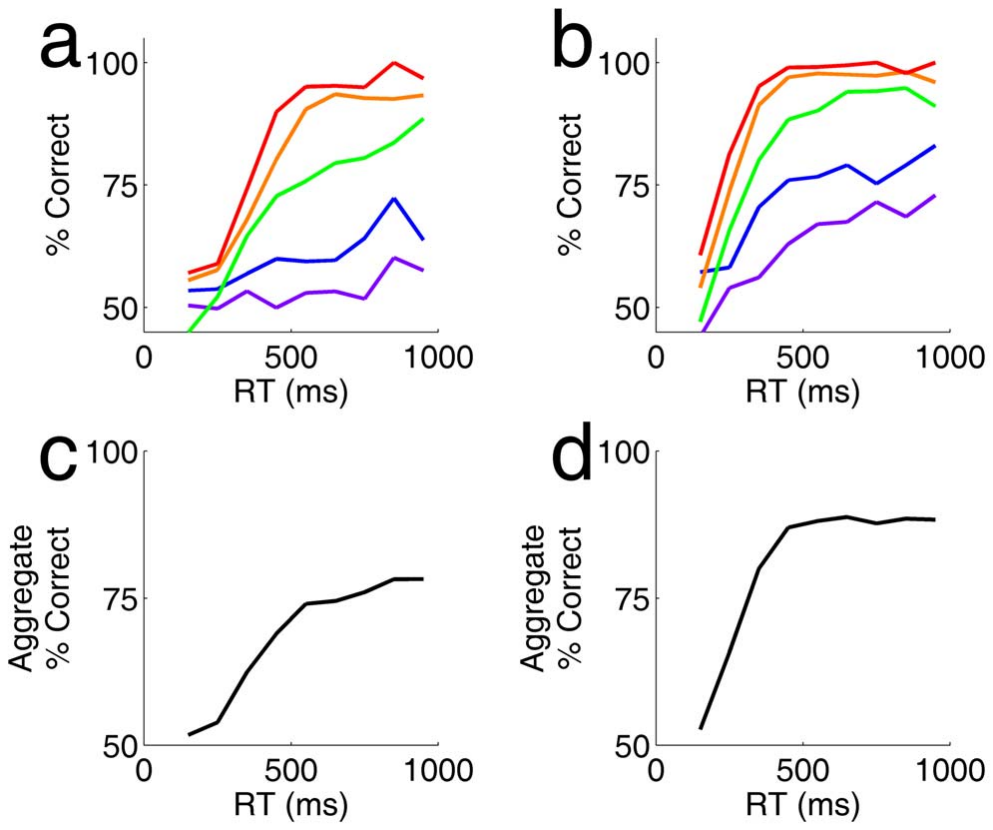
Accuracy improves with response time for each coherence, and overall. **a.** Accuracy (% correct) as a function of reaction time for different coherence ranges, from the experiment of Figure 3a. **b.** Accuracy as a function of reaction time from the experiment of Figure 3b. **c.** Aggregate accuracy as a function of reaction time combining all coherences, for the experiment analyzed in panel a. **d.** Aggregate accuracy as a function of reaction time for the experiment analyzed in panel b.

Reaction time increased with stimulus difficulty, while accuracy decreased. These two factors interact to determine the overall accuracy of rats as a function of time in an experiment with randomly interleaved coherence values. We found that accuracy increased with response time for any given coherence within the randomly interleaved trials, and the slope of this curve increased with coherence (Figure 4a,b). When all trials were combined, overall accuracy increased with reaction time (Figure 4c,d).

**Figure 5:**
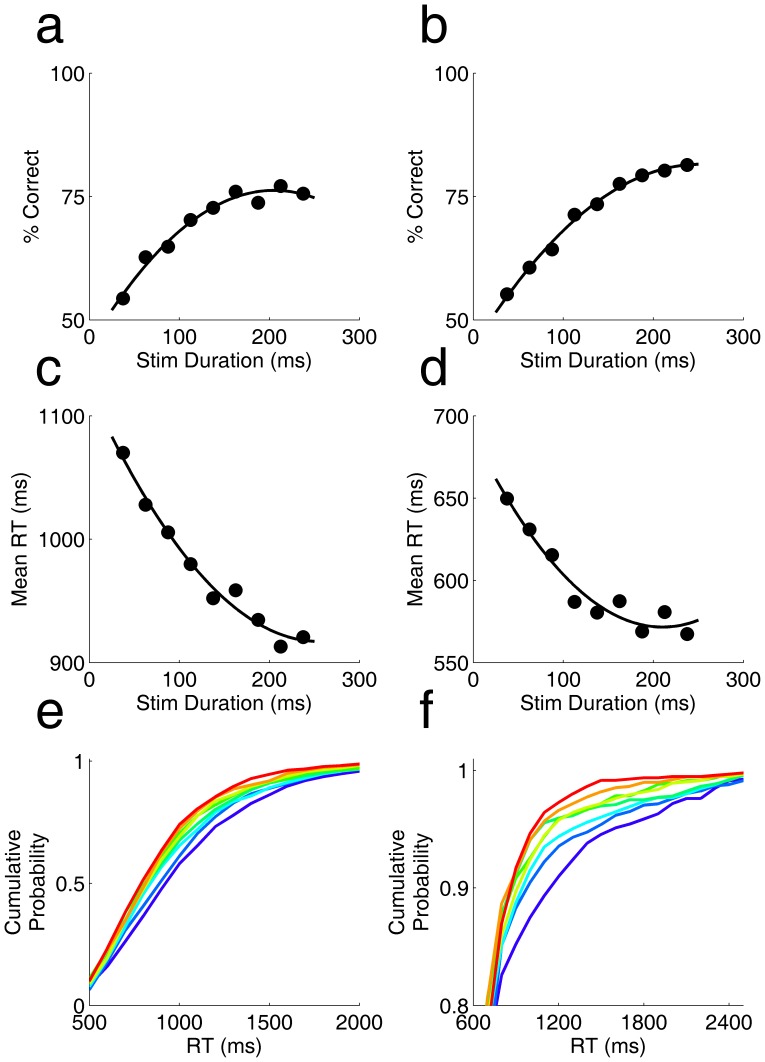
Dependence of accuracy and reaction time on stimulus duration. **a.** Accuracy (% correct) as a function of stimulus duration, from an experiment in which coherence was fixed at 85% and duration varied from 25 ms –225 ms in randomly interleaved trials. **b.** Accuracy as a function of motion strength for a second rat tested under same conditions as in a. **c.** Mean reaction time as a function of stimulus duration, for the experiment of panel a. **d.** Mean reaction time as a function of stimulus duration, for the experiment of pane b. **e.** Cumulative probability of reaction times for different stimulus durations from same data as panel a, with duration color coded from warm (long) to cool (brief). Cumulative probability was computed over the range of 0–3 s; axis is expanded to focus on the time range of interest. **f.** Cumulative probability of reaction times for different stimulus durations from data shown in panel b.

Above (Figures 1, 2 and 4) we showed that rats’ accuracy for a given coherence improves with reaction time. In those experiments, the stimulus duration was controlled by the rat’s response time. To uncouple these, we performed another experiment in which the termination of the trial was under control of the rat, but the time of stimulus offset was predetermined. The stimulus duration was chosen randomly in each trial from a uniform distribution from 25 to 225 ms, holding coherence fixed at 85%. Thus stimulus offset preceded the rat’s response for all but the fastest reaction times. We found that accuracy increased with stimulus duration over this range (Figure 5a,b). We note that rats responded later to more difficult (briefer) stimuli (Figure 5c–f) – even though the stimulus was no longer present.

**Figure 6:**
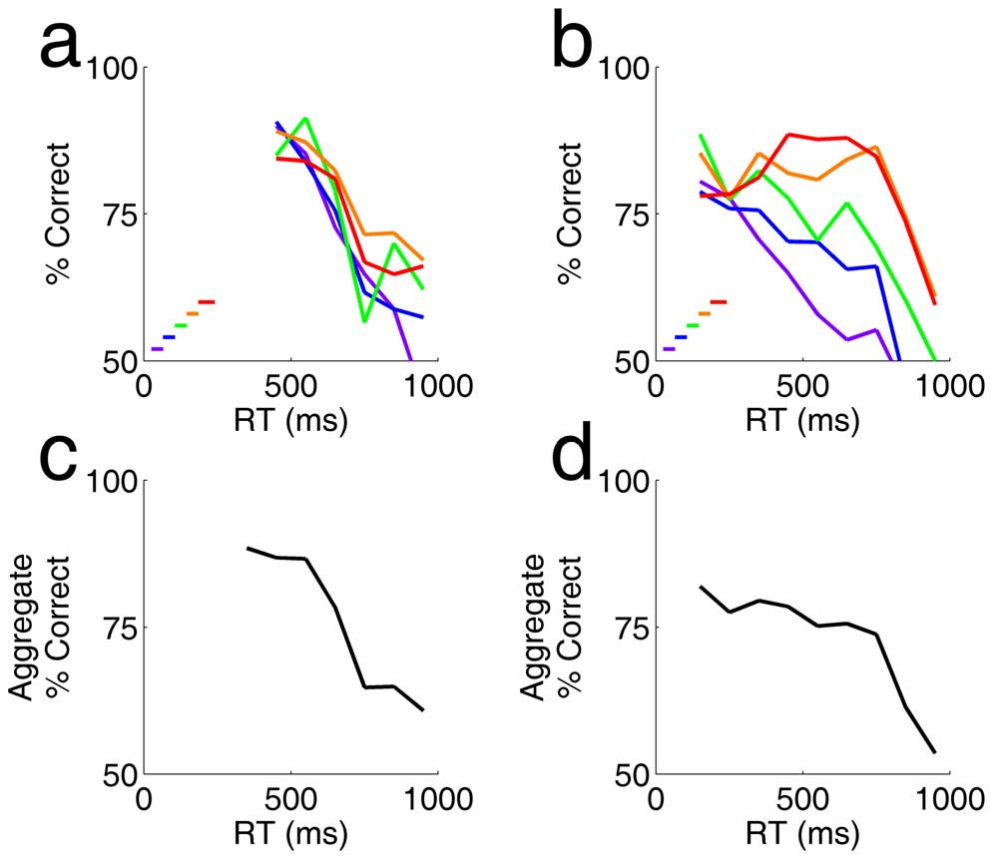
Accuracy decreases with response latency after stimulus offset. **a.** Accuracy (% correct) as a function of reaction time for one subject in a block of trials in which stimulus duration varied from 25–225 ms (same experiment as Figure 5a). Bars (lower left) show the range of stimulus durations that contributed to the curves of same color. **b.** Accuracy as a function of reaction time for a second rat (same experiment as Figure 5b). **c.** Aggregate accuracy as a function of reaction time, pooling together data from all stimulus durations, for the experiment analyzed in a. **d.** Aggregate accuracy as a function of reaction time for the experiment analyzed in b.

After stimulus offset, rats’ accuracy decreased with time (Figure 6), over the same range of reaction times for which we found an increase in accuracy when the stimulus was present (Figures 2, 4). This was the case for each stimulus duration considered separately (Figure 6 a,b) and in the experiment overall (Figure 6c,d).

## Discussion

We studied a self-paced motion discrimination task in which subjects could view the stimulus for any amount of time before committing to a response. We found that rats responded within a few seconds, and accuracy was positively correlated with reaction time for every rat and stimulus condition tested (Figure 1 b,c and Figure 2b).

Fast guessing in a subset of trials can produce such a correlation, but it is unlikely that this underlies our basic finding. Some rats showed a small peak of reaction times near their absolute motor delay (Figure 1 a,b: shoulder around 500 ms), at which time performance was at chance. But this peak accounted for a small fraction of trials at most; many rats lacked any discernable peak of fast responses. Moreover, accuracy increased continuously with reaction time across the range of reaction times observed (Figure 2a, Figure 4). Accuracy increased more steeply with reaction time for higher coherence stimuli (Figure 4 a,b), indicating that the rate of accumulation of usable evidence by the rat was determined by the rate of information in the stimulus itself.

Rats responded later to stimuli with lower coherence (Figure 3), indicating that their decision of when to commit to a response is sensitive to the quality of the sensory signal. This delay was small, however, compared with the difference in the rate of accumulation of evidence (Figure 4 a,b). In one experiment, for example, the reaction time at which accuracy reached 70% differed by 514 ms from the lowest to highest coherence (Figure 4b), while the observed reaction time changed by only 37 ms (Figure 3d). Therefore it is unlikely that the reaction time is explained by the time required to reach an evidence criterion [28], [29]. We suggest instead that reaction time was dominated by the cost of elapsed time [14], [30], [31].

When trials of all coherence were combined, we found that accuracy increased with reaction time in the experiment overall (Figure 4 c,d). This result differs from that previously reported for other coherent motion discrimination tasks with randomly interleaved coherence levels. In previous studies, accuracy decreased with reaction time overall (for example, see Figure 4 of [30]). The previous result could be explained if later reaction times were dominated by the lower coherence trials [30], [31]. In our task, however, low-coherence trials were only slightly more prevalent at later reaction times, so the aggregate result is dominated by the increase in accuracy with time within each coherence.

Our data are consistent with the idea that accuracy was determined by the amount of visual evidence that had accumulated at the time the rat committed to a response. But we considered the alternative possibility that rats entered some trials in a more attentive (or less impulsive) state than others. Such an unobserved internal state could cause the rat to have both higher accuracy and longer reaction times in the same trials, irrespective of the stimulus viewing time. On the evidence accumulation hypothesis, the improvement in accuracy with time should depend on the presence of the stimulus; any response delay after stimulus offset should not increase accuracy further. On the attentive trial hypothesis, the positive correlation between reaction time and accuracy is unrelated to viewing time and should exist regardless of stimulus duration.

Using limited duration stimuli, we found that after stimulus offset, there was no further increase in accuracy with reaction time (Figure 6). This supports the interpretation that later responses were more accurate in our first experiment because more visual evidence had accumulated by the time the trial was terminated, rather than because of a different internal state of the animal. Indeed we found a marked decrease in accuracy with reaction time after stimulus offset. This suggests that in the absence of visual signal, noise accumulates with time in the rats’ estimate of motion direction.

Rats responded later on more difficult trials in the limited duration experiment (Figure 4 c,d) – even though the stimulus was absent, and the delay only impaired performance (Figure 5 c,d). This suggests that in the unlimited viewing condition, the dependence of reaction time on trial difficulty may be related to the rat’s confidence [32], [33], rather than any direct signature of the rate of time-dependent evidence accumulation.

The main difference between our results and previous reports is that in our study, elapsed time appeared to be more important than an evidence criterion in determining the subjects’ choice of when to terminate trials. This difference could be due to species (rat vs. primate), but might well be explained by differences in the temporal structures of the tasks. In the previous studies, in addition to the penalty time out periods, there was generally a delay after trial initiation and before choice targets, another (often variable) delay before stimulus onset, an enforced minimum delay after stimulus onset before reward delivery, and/or an enforced inter-trial interval after both correct and incorrect responses. These delays, which often added several seconds to the duration of a trial, were imposed specifically to discourage fast responses.

In our task, delays were imposed only for error penalties, and fast responses were explicitly allowed. The elapsed time during decisions could therefore be a substantial fraction of the total time, and thus might weigh more heavily in subjects’ decisions. A moderately confident subject in our task could harvest reward instantly and start a new trial immediately if he is correct; waiting for more evidence would only pay off if the incremental reduction in the risk of error offsets the opportunity cost of the delay. It is possible that our rats optimally balanced the expected value of information against the cost of time. Future experiments could explicitly test this hypothesis.

## Materials and Methods

### Ethics

All procedures were conducted with care to avoid any pain or suffering in animal subjects. This work was conducted in an AAALAC-accredited facility with the approval and under the supervision of the Institutional Animal Care and Use Committee at the University of California San Diego.

### Behavioral Training and Testing Overview

Six male Long-Evans rats (Harlan) were water restricted and trained to perform visual tasks for water reward (Meier, Flister & Reinagel 2011). Subjects began training at age p30 for 2 hrs/day 7 days a week. Subjects performed 500–1500 trials per day, and received water in 50% of trials when performing at chance. No supplemental water was given except on rare occasions when a training day was skipped. Hydrating treats (carrots) were given after each training session. During training sessions subjects had free access to return to the home cage at any time; thus they had access to food during periods of water consumption. On this protocol, all subjects maintained normal growth curves (within 5% of published values for unrestricted food and water). Between training sessions, subjects were pair-housed with enrichment (chew toys, PVC tubes). Subjects were housed in a reverse 12 hour light/dark cycle and were trained and tested during the dark cycle.

Animals were trained to perform two alternative forced choice (2 AFC) visual discrimination of the direction of motion in a random dot display. Each trial was initiated by the subject by licking a central request port, which caused the motion stimulus to appear immediately on a CRT monitor in front of the rat. The response port toward which the majority of dots moved was associated with reward (water) and the other response port was associated with penalty (time-out). In experiments of Figures 1, 2, 3, 4, the visual stimulus persisted until the subject licked a response port (L or R), with no time limit. In the experiments described in Figures 5 and 6, the stimulus had a predetermined duration followed by a blank screen until the subject responded. After correct responses, water was delivered at the response location with <10 ms delay, after which the subject could immediately initiate a new trial. After errors, a darkened screen indicated a timeout penalty before a new trial could be initiated. The end of the timeout was indicated by return to the start screen (mean luminance). The time required to learn the task varied considerably across subjects (Table 1). Two additional subjects were removed from the study for failure to reach criterion performance in 30 days.

**Table 1:** Task Acquisition.

Rat	Days to Criterion	Trials to Criterion
1	10	1953
2	19	2507
3	5	4037
4	9	6789
5	22	13220
6	15	9623

All subjects were trained with 85% coherent motion. Time required to reach criterion (80% correct performance) is measured in calendar days (with training every day) or in trials (all trials performed, including correction trials).

Reward magnitude (water volume) was empirically adjusted for each rat to ensure adequate hydration and normal growth curve, while maximizing motivation as judged by the number of trials per day. Penalty time out duration was empirically adjusted for each rat to discourage guessing, while avoiding excessive subject frustration as judged by quitting. Both reinforcement parameters remained fixed for each rat within each training session, and were adjusted infrequently over the rat’s lifespan.

Automated correction trials were used throughout training and testing to prevent and correct bias and perseveration. After every error trial, the following trial had a 20% probability of being a correction trial. In a correction trial, all parameters of the stimulus and trial were set, or selected randomly, exactly as in a normal trial. But the direction of motion was set deterministically to the same side as the last trial – the side the rat just failed to choose. After all the other error trials, and after all correct trials, left and right directions of motion direction were chosen with equal probability, and the motion direction was selected randomly and independently in each trial. Note that long runs of the same side are not rare in random binary sequences, and were not avoided.

### Details of Tasks and Data Analysis

Data analysis was performed using Matlab (Mathworks, Natick MA). Each behavioral testing block is defined by the statistics of the stimulus ensemble (such as the distribution of coherence values), as well as the magnitude of reward on correct trials and the duration of the time-out on error trials. Each single trial is further defined by the specific visual stimulus (selected from the ensemble independently each trial) and the rewarded side (selected independently each trial, except for correction trials, which are therefore excluded from analysis). In each trial we recorded these variables as well as the time of subject-initiated stimulus request, the latency from stimulus onset to response, and the outcome of the trial (correct/reward or error/timeout).

Data were taken for analysis only after training to asymptotic performance in that block condition, and during blocks within which performance was stationary. Only some trials after errors were correction trials, but all trials after errors were excluded from analysis. All remaining trials with reaction times between 0–3000 ms were included; of these fewer than 1% of trials had reaction times longer than 2000 ms.

In all these experiments, the stimuli were displayed on a CRT monitor 10 cm from the rat’s eye, subtending about 104° of visual angle with a resolution of 0.1 degrees/pixel, All stimuli were presented at 100% contrast, and with a density of100 dots uniformly distributed on the entire display. As individual dots moved off the edge of the display, each was replaced with a dot at a new random location.

Six rats participated in the experiments analyzed in Figures 1 and 2. Data were taken from testing blocks containing only a single coherence. In the example shown in Figures 1a, 1b and 2a, the analysis is based on 3,721 trials (2,641 correct trials and 1,080 error trials). Across the 6 rats and multiple task variants included in Figure 1 c and d and Figure 2b, the number of trials contributing to the analysis ranged from 3,271 to 10,693. The dot size, speed and coherence differed in the different experiments.

Two rats participated in the experiments analyzed in Figures 3 and 4. In these experiments, coherence was varied from trial to trial in a randomly interleaved protocol. Prior to testing, two subjects were trained in blocks of progressively more difficult coherence ranges (70–90%; 50–90%; 30–90%; 15–45%) for a total of at least 16,000 trials of practice in coherent dot discrimination. Subjects were then tested in blocks with different stimulus distributions, of which representative examples are shown. In the experiment analyzed in Figures 3a, 3c, 4a, 4c, and 4e, coherence was distributed uniformly over a continuous range from 0–100%; the contrast (100%), dot size (1.8 degrees visual angle in diameter) and speed (60 degrees per second) were held constant. A total of 16,076 trials contributed to these analyses. In the experiment analyzed in Figures 3b, 3d, 4b, 4d, and 4e, coherence ranged uniformly over the range 20–90%, contrast was fixed at 100%. Dot size (from 0.3–3.0 degrees visual angle) and speed (from 60–180 degrees per second) were also randomly and independently chosen each trial. We found no difference in performance or reaction time as a function of dot size or speed (analysis not shown) so these data were pooled, for a total of 29,261 trials contributing to the analysis shown. Note that coherence was binned more coarsely in Figure 4 than in Figure 3, in order to provide enough trials to further subdivide the trials by reaction time. Color scale is consistent within each figure but differs between figures.

Two rats participated in the experiments analyzed in Figures 5 and 6. In these experiments, coherence (85%), contrast (100%), dot size (1.8 degrees visual angle in diameter) and motion speed (60 degrees per second) were held constant. The duration of the stimulus was randomly selected uniformly on the range 25 ms to 250 ms. The stimulus onset occurred immediately upon trial request in each case, and ended at the predetermined time regardless of the rats’ behavior. The probability of stimulus duration was uniform on the interval we used, so the probability of offset increased with time during the brief stimulus presentation. Rats could in principle anticipate the stimulus offset during this short time. They did not have to wait for stimulus offset or a “go signal” before responding, however; nor were they required to respond within any time limit after the stimulus offset. The data do not indicate that the response time was triggered by the stimulus offset; to the contrary, the briefest movies had the longest reaction times.

Note that duration was binned more coarsely in Figure 6 than in Figure 5, in order to provide enough trials to further subdivide the trials by reaction time; the color scale is consistent within each figure but differs between figures. Most stimuli were briefer than the rats’ earliest responses, but for the longest stimuli the rat may have committed to a response while the stimulus was still present. This is particularly the case for the subject in panels 5b, 5d, 5b and 6d, which had a short absolute motor delay due to the use of response ports so close to the request port (+/−10 mm) that he could complete an entire trial without moving his head.
